# Supplementary material for: The Association between Peptic Ulcer Disease and Gastric Cancer: Results from the Stomach Cancer Pooling (StoP) Project Consortium
Source: Cancers (Basel). 2022 Oct 7;14(19):4905. doi: 10.3390/cancers14194905 (PMC9563899; doi:10.3390/cancers14194905)
Supplement: Supplementary file 1 [file cancers-14-04905-s001.zip › cancers-1937174-supplementary.pdf]

Supplementary Table S1. Characteristics of the Participating Studies in the Stomach Cancer Pooling (StoP) Consortium with Available Data on Gastric or Duodenal Ulcer

Study	Cases n (%)	Controls n (%)	Type of Control	Age at Entry (Mean±SD)	Male n (%)	Peptic Ulcer n (%)	Gastric Ulcer n (%)	Duodenal Ulcer n (%)	Missing PUD data n (%)
**Italy 1 (La Vecchia et al., 1995)**	769 (27.0)	2,081 (73.0)	Hospital	55.5±11.6	1,689 (59.3)	299 (10.5)	156 (5.5)	167 (5.9)	1 (0.04)
**Italy 3 (De Feo et al., 2012)**	160 (26.5)	444 (73.5)	Hospital	61.2±15.3	345 (57.1)	6 (1.0)	5 (0.8)	1 (0.2)	9 (1.5)
**Italy 4 (Buiatti et al., 1989)**	1,016 (46.7)	1,159 (53.3)	Population	63.3±9.7	1,345 (61.8)	308 (14.2)	145 (6.7)	188 (8.6)	0 (0.0)
**Greece (Lagiou et al., 2004)**	110 (52.4)	100 (47.6)	Hospital	62.3±12.2	106 (50.5)	—	—	34 (16.2)	0 (0.0)
**Russia (Zaridze et al., 2000)**	450 (42.4)	611 (57.6)	Hospital	58.3±11.3	541 (51.0)	101 (9.5)	69 (6.5)	49 (4.6)	6 (0.6)
**China 1 (Deandrea et al., 2010)**	266 (33.3)	533 (66.7)	Hospital	55.1±10.1	618 (77.4)	—	38 (4.8)	—	0 (0.0)
**China 2 (Mu et al., 2005)**	206 (33.2)	415 (66.8)	Population	59.0±11.3	425 (68.4)	—	60 (9.7)	—	0 (0.0)
**China 3 (Setiawan et al., 2005)**	711 (50.0)	711 (50.0)	Population	62.6±11.5	906 (63.7)	159 (11.2)	105 (7.4)	58 (4.1)	188 (13.2)
**China 4 (Setiawan et al., 2001)**	133 (24.0)	433 (77.0)	Population	49.8±12.3	307 (54.2)	46 (8.1)	20 (3.5)	29 (5.1)	45 (8.0)
**Japan 3 (Machida-Montani**	153 (33.5)	303 (66.5)	Hospital	57.8±9.4	323 (70.8)	101 (22.2)	74 (16.2)	45 (9.9)	0 (0.0)

**et al., 2004)**									
**USA 1 (Zhang et al., 1999)**	132 (50.0)	132 (50.0)	Hospital	57.7±13.9	156 (59.1)	—	91 (34.5)	—	18 (6.8)

SD: standard deviation; n: count; PUD: Peptic Ulcer Disease

** had 42% of controls selected from the population but considered as hospital-controlled for the stratified analysis by control type.

— Data not available or incomplete for the study or region

Supplementary Table S2. Distribution of Peptic ulcer types in gastric cancer cases and controls according to Study in the Stomach Cancer Pooling (StoP) Consortium with Available Data on Gastric or Duodenal Ulcer

Study	Gastric Ulcer		Duodenal Ulcer	
	Gastric Cancer Cases with Gastric Ulcer n (%)	Controls with Gastric Ulcer n (%)	Gastric Cancer Cases with Duodenal Ulcer n (%)	Controls with Duodenal Ulcer n (%)
**Italy 1 (La Vecchia et al., 1995)**	74 (9.6)	82 (4.0)	58 (7.5)	109 (5.2)
**Italy 3 (De Feo et al., 2012)**	3 (1.9)	2 (0.5)	1 (0.6)*	0 (0.0)
**Italy 4 (Buiatti et al., 1989)**	95 (9.4)	50 (4.3)	85 (8.4)	103 (8.9)
**Greece (Lagiou et al., 2004)**	—	—	19 (17.3)	15 (15.0)
**Russia (Zaridze et al., 2000)**	53 (11.8)	16 (2.6)	32 (7.1)	17 (2.8)
**China 1 (Deandrea et al., 2010)**	32 (12.0)	6 (1.1)	—	—
**China 2 (Mu et al., 2005)**	43 (20.9)	17 (4.1)	—	—
**China 3 (Setiawan et al., 2005)**	91 (12.8)	14 (2.0)	39 (5.5)	19 (2.7)
**China 4 (Setiawan et al., 2001)**	5 (3.8)	15 (3.5)	3 (2.3)	26 (6.0)
**Japan 3 (Machida-Montani et al., 2004)**	36 (23.5)	38 (12.5)	16 (10.5)	29 (9.6)
**USA 1 (Zhang et al., 1999)**	55 (41.7)	36 (27.3)	—	—
**Total**	487 (12.5)	276 (4.1)	252 (7.8)	318 (6.0)

- Data not available or incomplete for the study or region

*Study Italy 3 was removed from the pooled analysis due to low number of cancer c
