# Supplementary figures and images for: The Association between Peptic Ulcer Disease and Gastric Cancer: Results from the Stomach Cancer Pooling (StoP) Project Consortium
Source: Cancers (Basel). 2022 Oct 7;14(19):4905. doi: 10.3390/cancers14194905 (PMC9563899; doi:10.3390/cancers14194905)

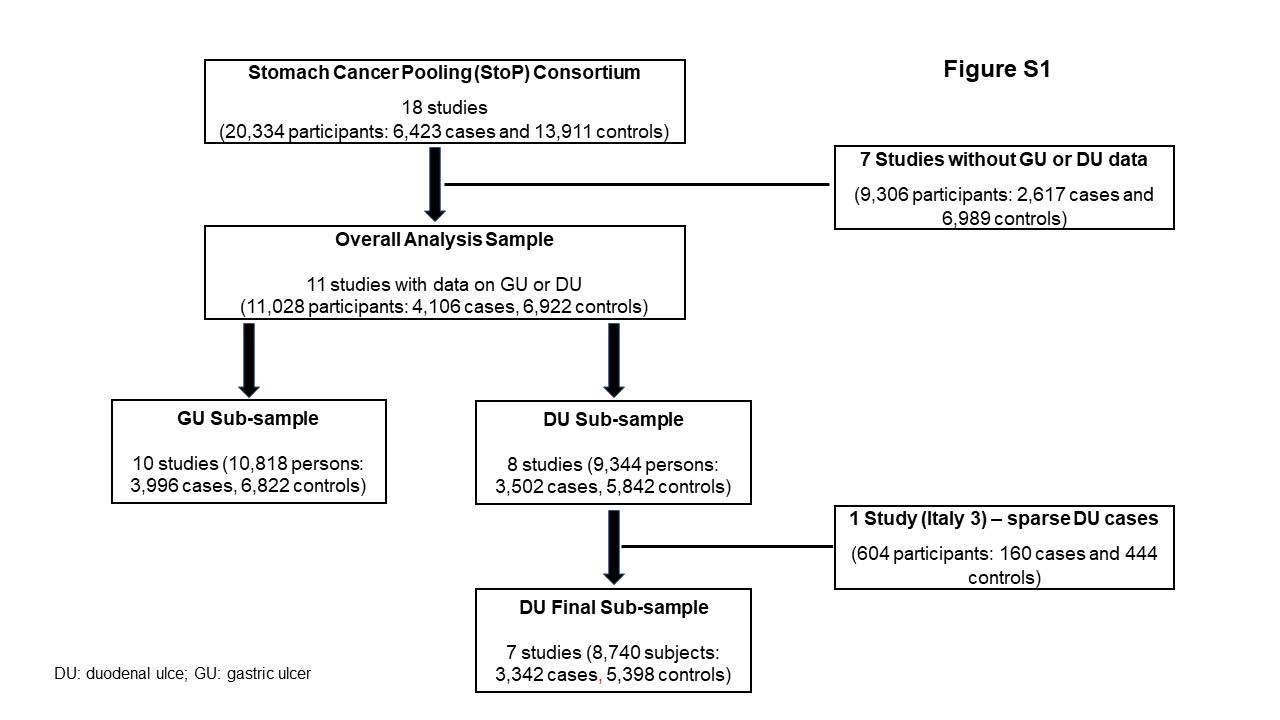

Supplement: Supplementary file 1 [file cancers-14-04905-s001.zip › cancers-1937174-supplementary.jpg]
